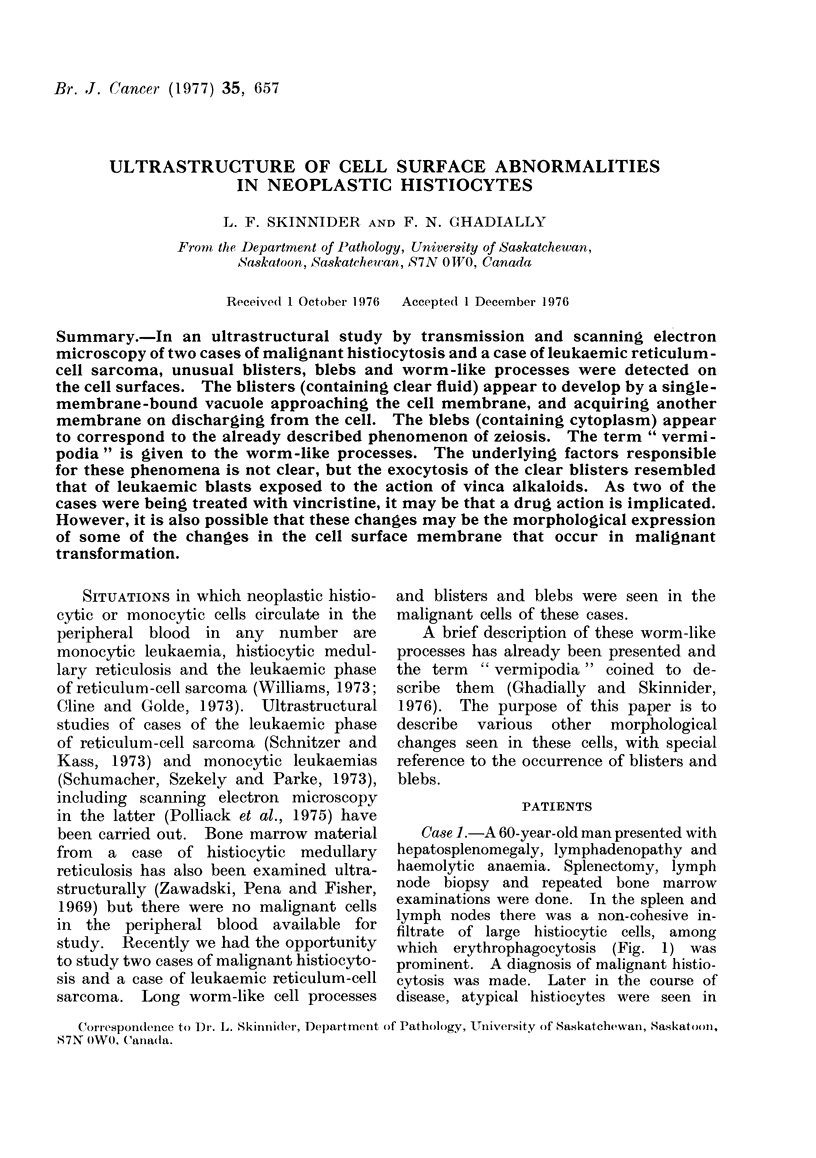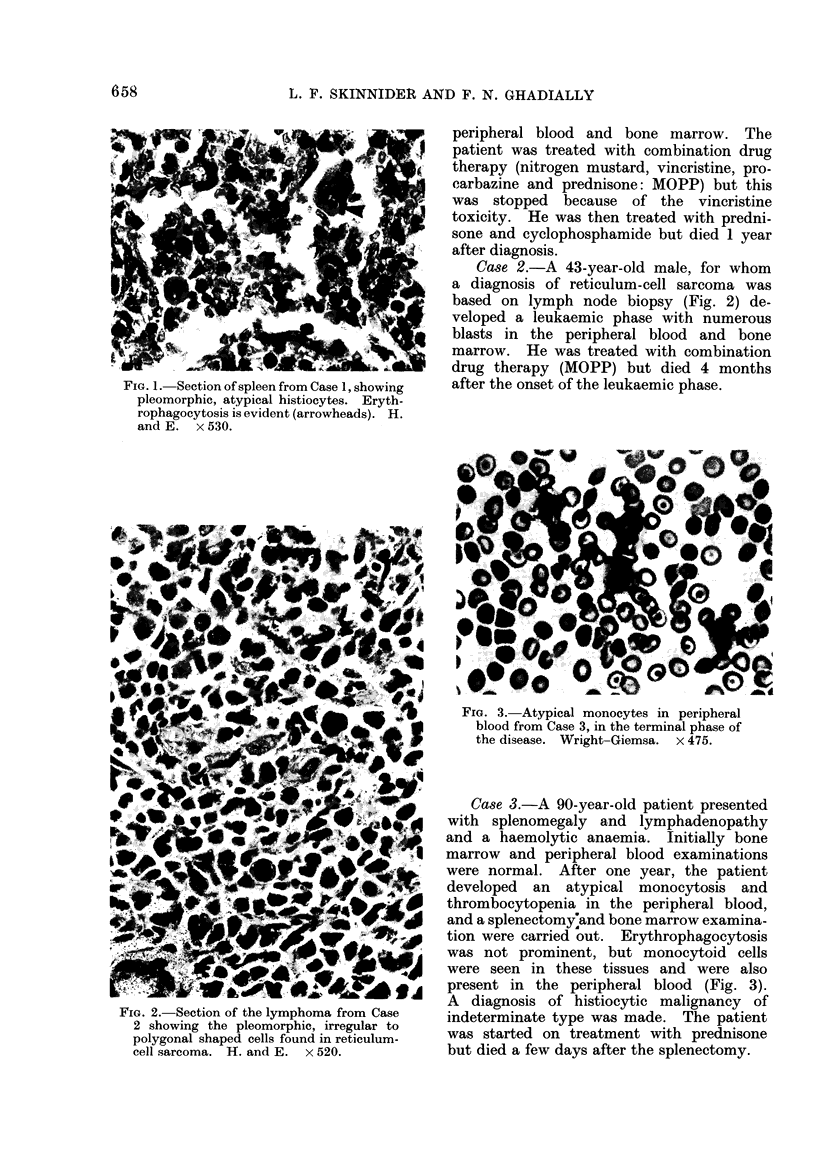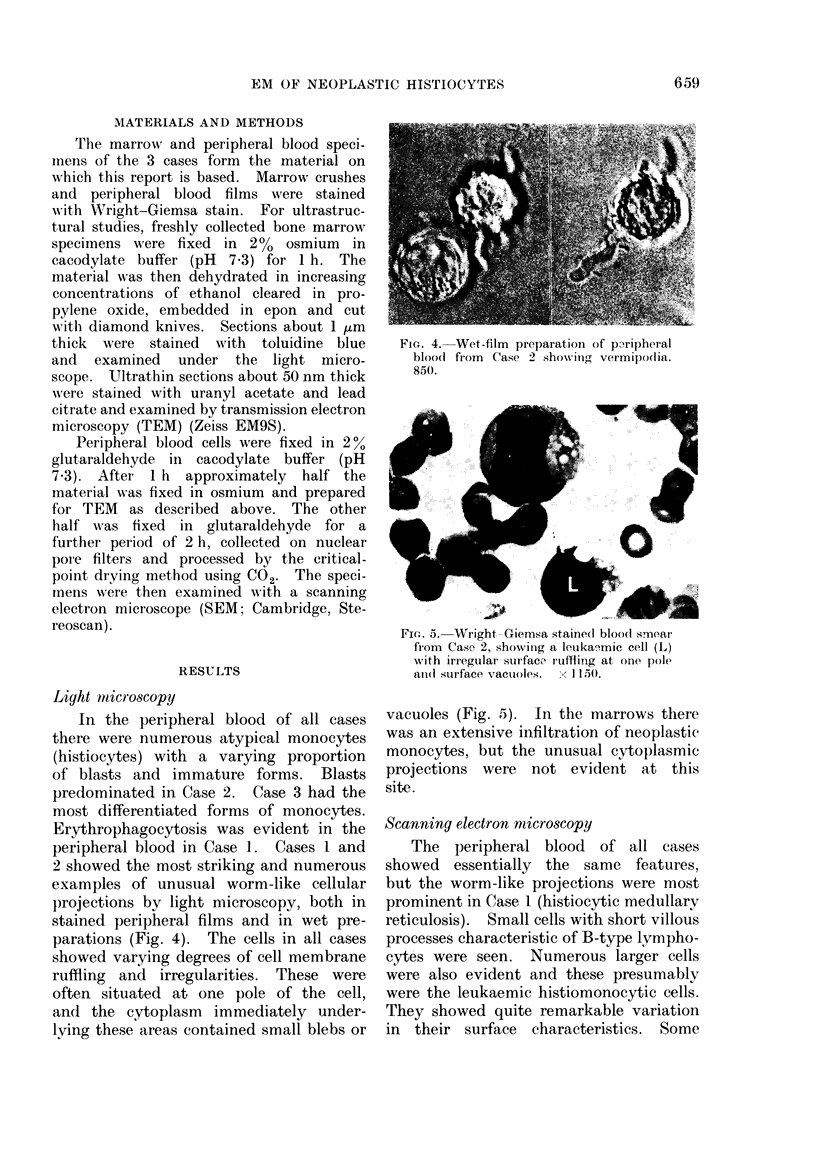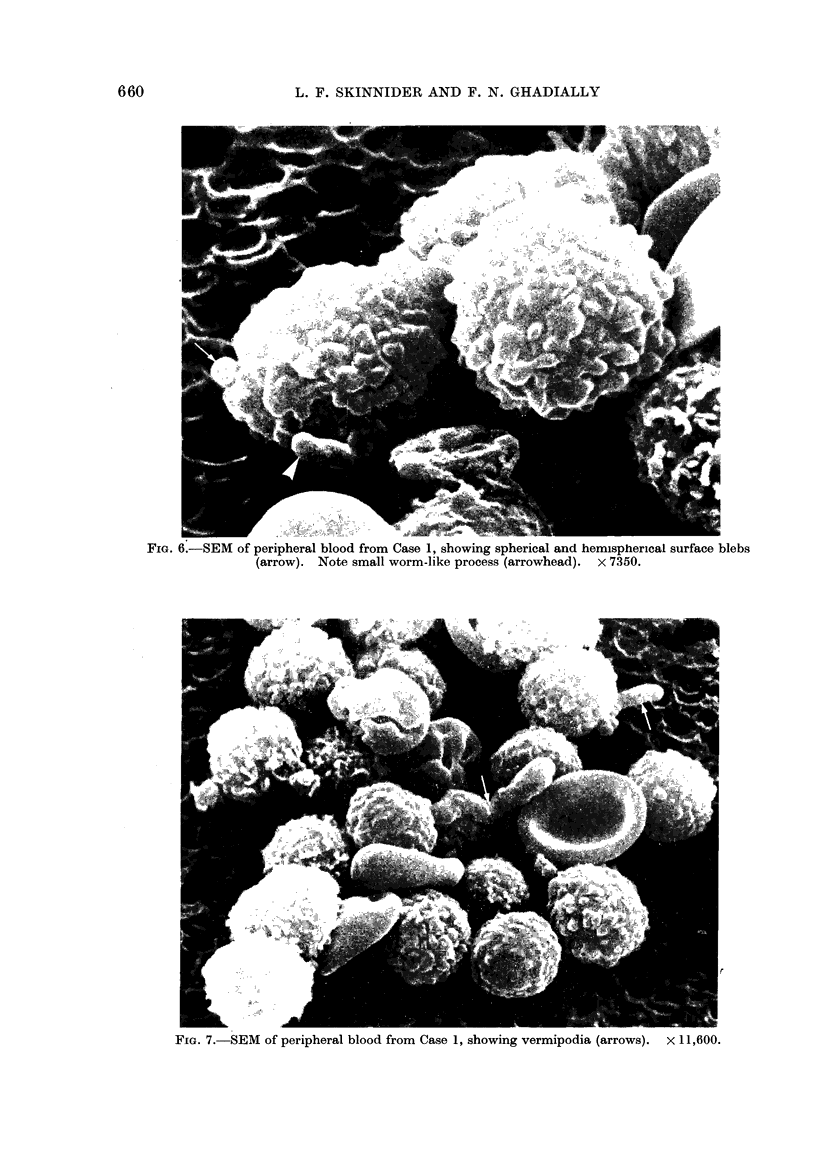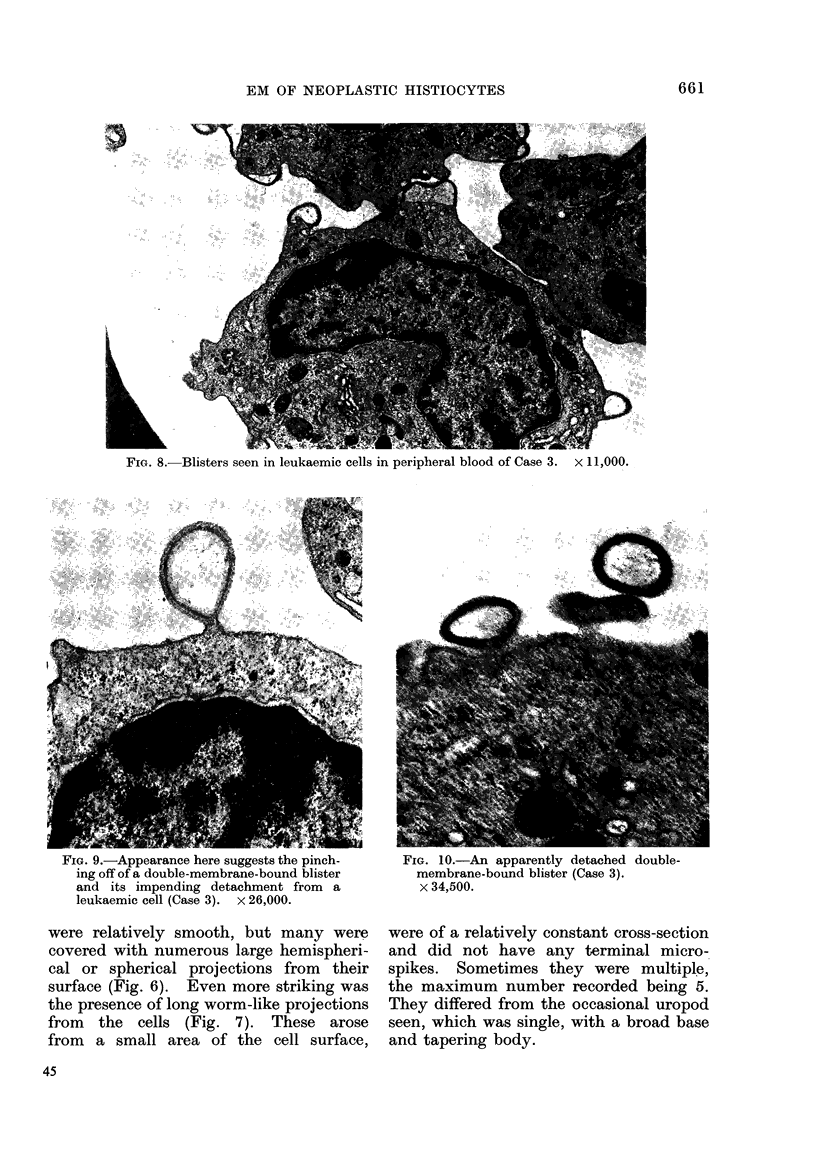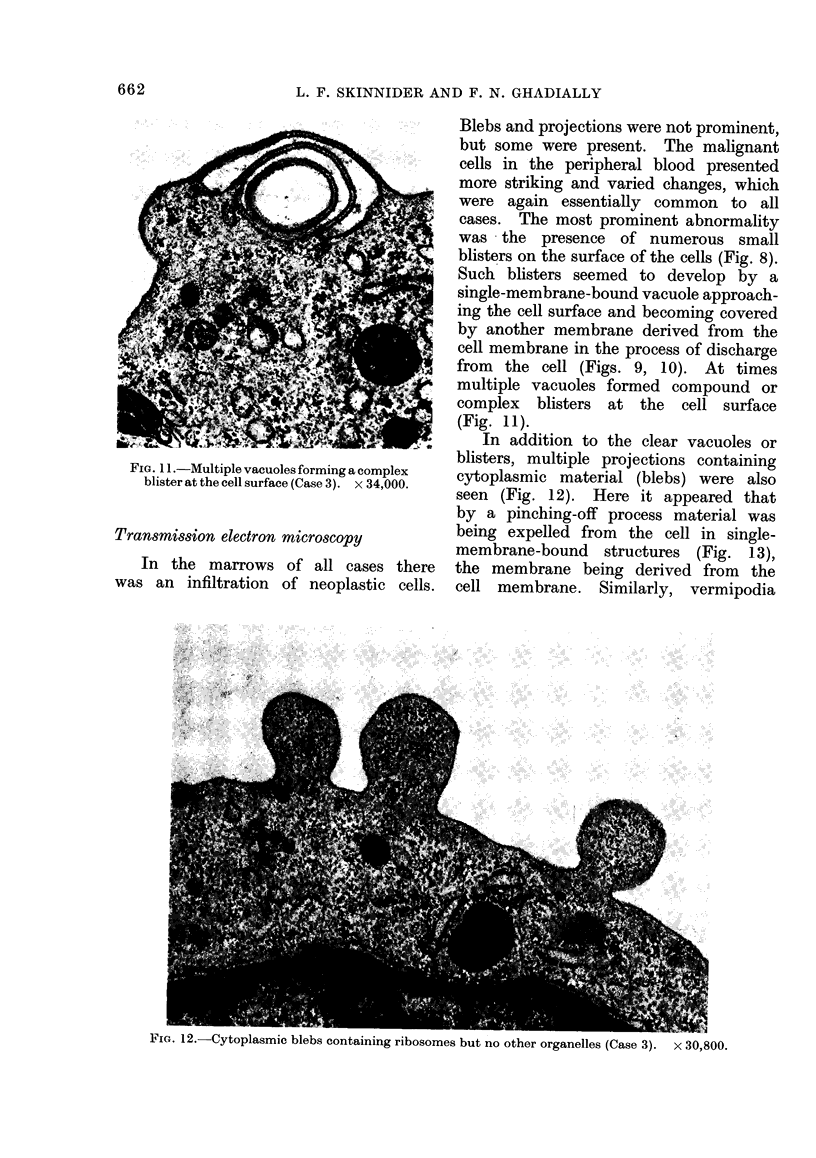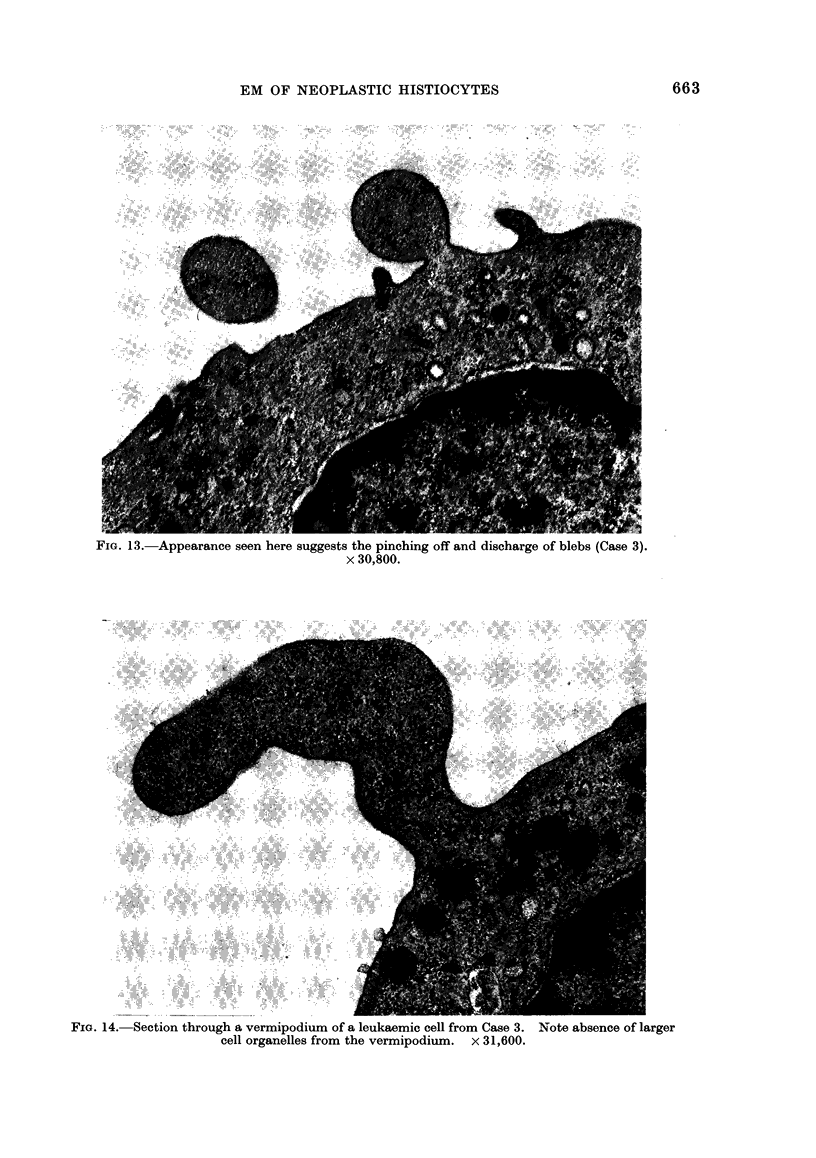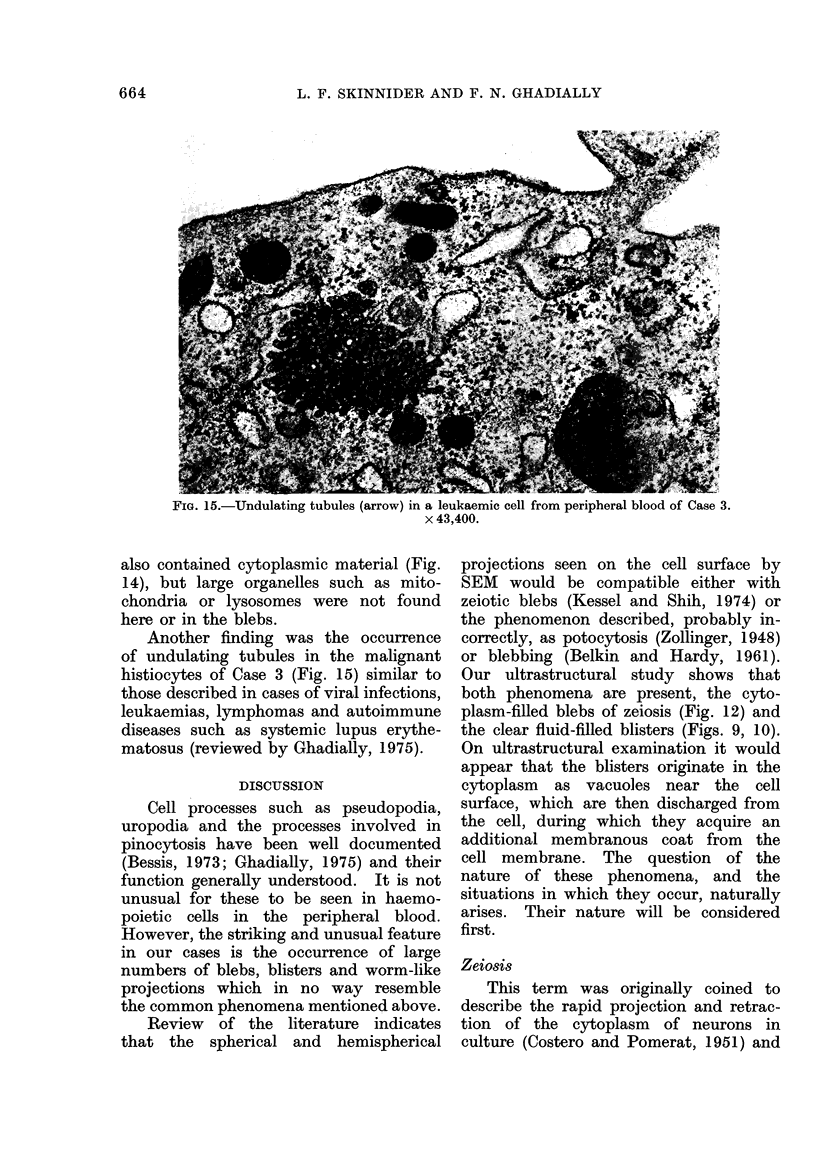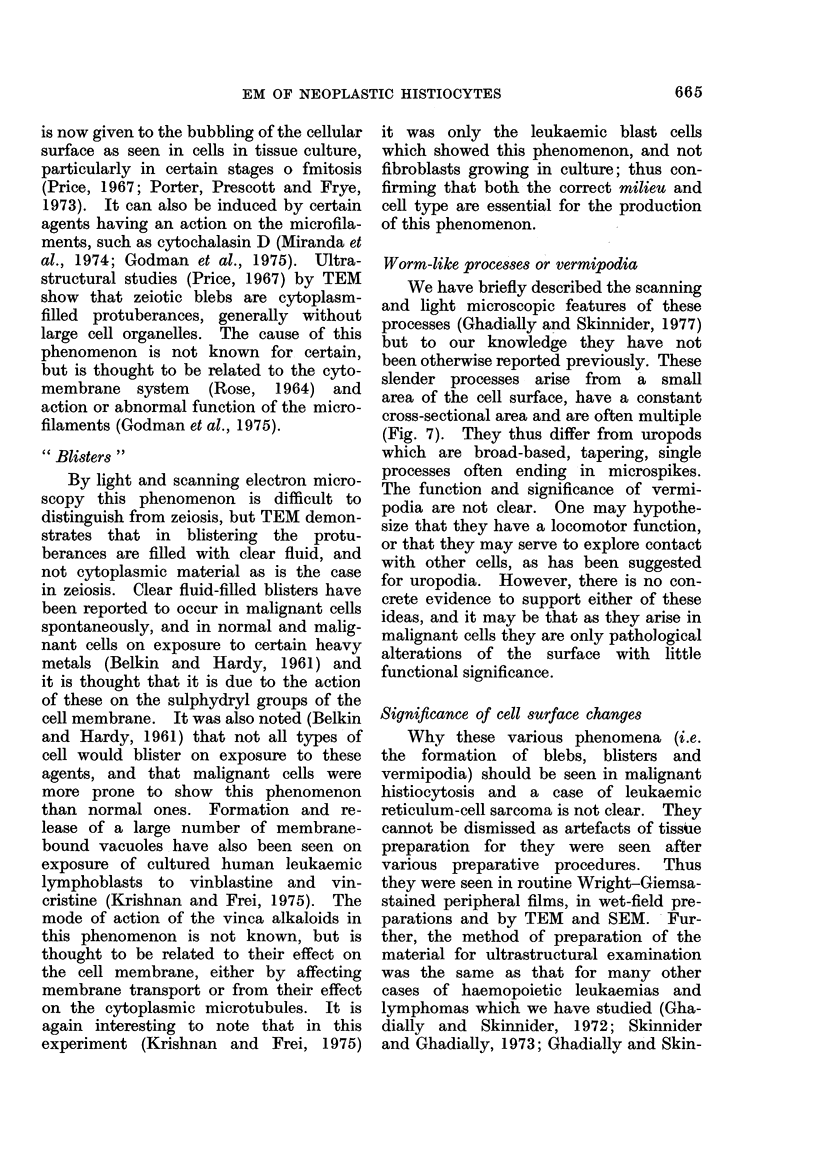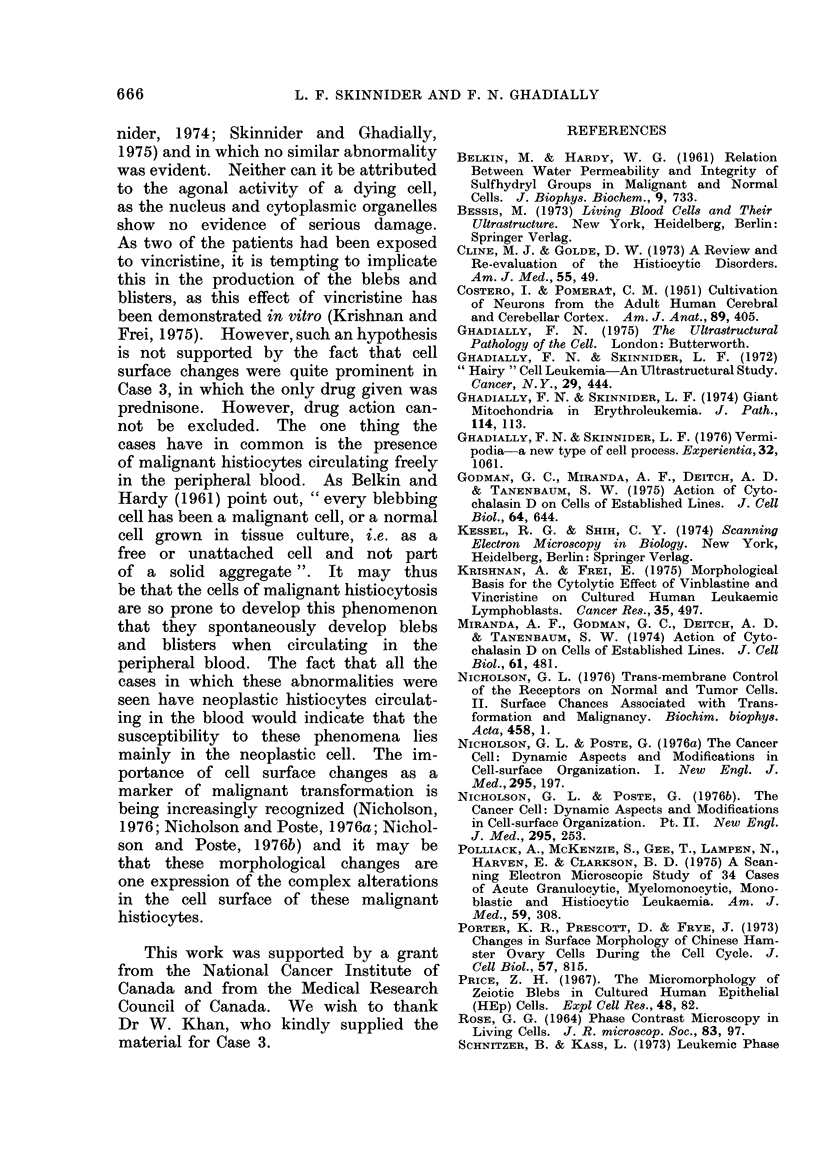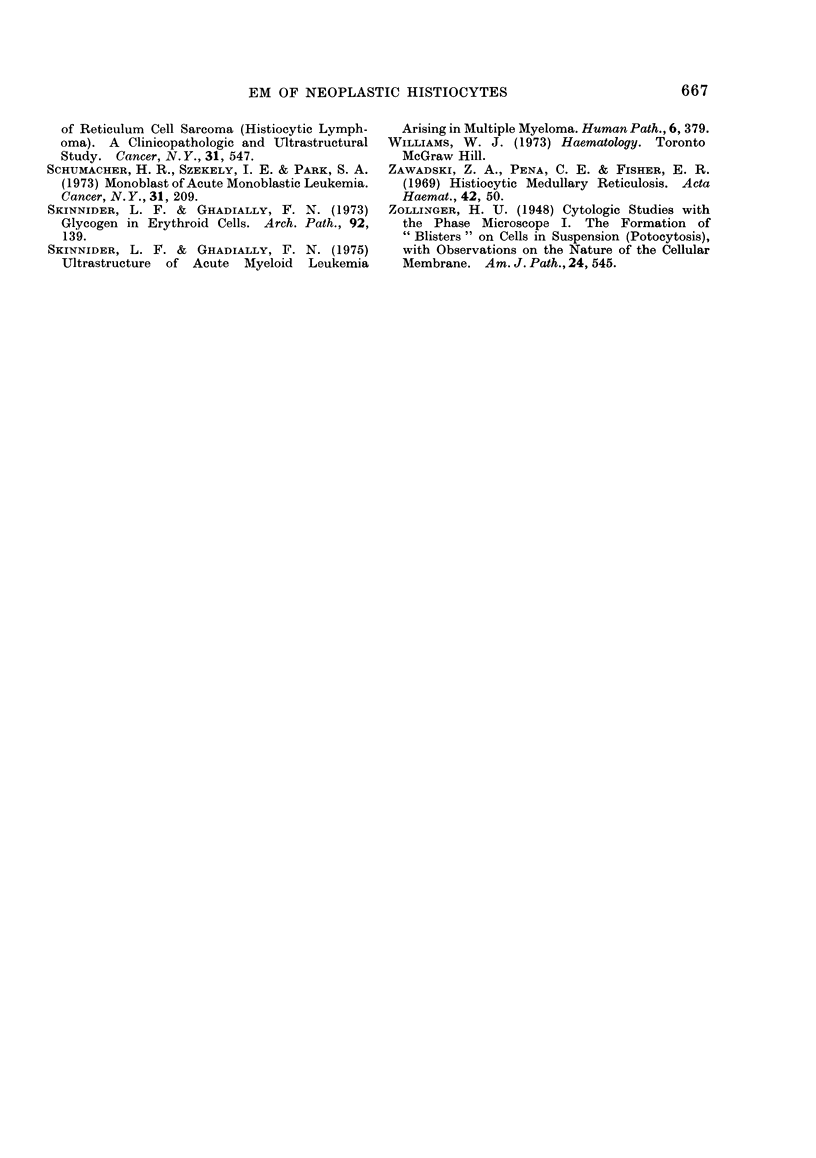# Ultrastructure of cell surface abnormalities in neoplastic histiocytes.

**DOI:** 10.1038/bjc.1977.101

**Published:** 1977-05

**Authors:** L. F. Skinnider, F. N. Ghadially

## Abstract

**Images:**


*Br. J. Cancer* (1977) **35**, 657

### ULTRASTRUCTURE OF CELL SURFACE ABNORMALITIES IN NEOPLASTIC HISTIOCYTES

L. F. SKINNIDER AND F. N. GHADIALLY

*From the Department of Pathology, University of Saskatchewan, Saskatoon, Saskatchewan, S7N 0W0, Canada*

Received 1 October 1976 Accepted 1 December 1976

**Summary.—In an ultrastructural study by transmission and scanning electron microscopy of two cases of malignant histiocytosis and a case of leukaemic reticulum-cell sarcoma, unusual blisters, blebs and worm-like processes were detected on the cell surfaces. The blisters (containing clear fluid) appear to develop by a single-membrane-bound vacuole approaching the cell membrane, and acquiring another membrane on discharging from the cell. The blebs (containing cytoplasm) appear to correspond to the already described phenomenon of zeiosis. The term " vermipodia " is given to the worm-like processes. The underlying factors responsible for these phenomena is not clear, but the exocytosis of the clear blisters resembled that of leukaemic blasts exposed to the action of vinca alkaloids. As two of the cases were being treated with vincristine, it may be that a drug action is implicated. However, it is also possible that these changes may be the morphological expression of some of the changes in the cell surface membrane that occur in malignant transformation.**

SITUATIONS in which neoplastic histiocytic or monocytic cells circulate in the peripheral blood in any number are monocytic leukaemia, histiocytic medullary reticulosis and the leukaemic phase of reticulum-cell sarcoma (Williams, 1973; Cline and Golde, 1973). Ultrastructural studies of cases of the leukaemic phase of reticulum-cell sarcoma (Schnitzer and Kass, 1973) and monocytic leukaemias (Schumacher, Szekely and Parke, 1973), including scanning electron microscopy in the latter (Polliack *et al.*, 1975) have been carried out. Bone marrow material from a case of histiocytic medullary reticulosis has also been examined ultrastructurally (Zawadski, Pena and Fisher, 1969) but there were no malignant cells in the peripheral blood available for study. Recently we had the opportunity to study two cases of malignant histiocytosis and a case of leukaemic reticulum-cell sarcoma. Long worm-like cell processes and blisters and blebs were seen in the malignant cells of these cases.

A brief description of these worm-like processes has already been presented and the term " vermipodia " coined to describe them (Ghadially and Skinnider, 1976). The purpose of this paper is to describe various other morphological changes seen in these cells, with special reference to the occurrence of blisters and blebs.

### PATIENTS

*Case 1.*—A 60-year-old man presented with hepatosplenomegaly, lymphadenopathy and haemolytic anaemia. Splenectomy, lymph node biopsy and repeated bone marrow examinations were done. In the spleen and lymph nodes there was a non-cohesive infiltrate of large histiocytic cells, among which erythrophagocytosis (Fig. 1) was prominent. A diagnosis of malignant histiocytosis was made. Later in the course of disease, atypical histiocytes were seen in

Correspondence to Dr. L. Skinnider, Department of Pathology, University of Saskatchewan, Saskatoon, S7N 0W0, Canada.

FIG. 1.—Section of spleen from Case 1, showing pleomorphic, atypical histiocytes. Erythrophagocytosis is evident (arrowheads). H. and E. ×530.

FIG. 2.—Section of the lymphoma from Case 2 showing the pleomorphic, irregular to polygonal shaped cells found in reticulum-cell sarcoma. H. and E. ×520.

peripheral blood and bone marrow. The patient was treated with combination drug therapy (nitrogen mustard, vincristine, procarbazine and prednisone: MOPP) but this was stopped because of the vincristine toxicity. He was then treated with prednisone and cyclophosphamide but died 1 year after diagnosis.

*Case 2.*—A 43-year-old male, for whom a diagnosis of reticulum-cell sarcoma was based on lymph node biopsy (Fig. 2) developed a leukaemic phase with numerous blasts in the peripheral blood and bone marrow. He was treated with combination drug therapy (MOPP) but died 4 months after the onset of the leukaemic phase.

FIG. 3.—Atypical monocytes in peripheral blood from Case 3, in the terminal phase of the disease. Wright–Giemsa. ×475.

*Case 3.*—A 90-year-old patient presented with splenomegaly and lymphadenopathy and a haemolytic anaemia. Initially bone marrow and peripheral blood examinations were normal. After one year, the patient developed an atypical monocytosis and thrombocytopenia in the peripheral blood, and a splenectomy and bone marrow examination were carried out. Erythrophagocytosis was not prominent, but monocytoid cells were seen in these tissues and were also present in the peripheral blood (Fig. 3). A diagnosis of histiocytic malignancy of indeterminate type was made. The patient was started on treatment with prednisone but died a few days after the splenectomy.

MATERIALS AND METHODS

The marrow and peripheral blood specimens of the 3 cases form the material on which this report is based. Marrow crushes and peripheral blood films were stained with Wright–Giemsa stain. For ultrastructural studies, freshly collected bone marrow specimens were fixed in 2% osmium in cacodylate buffer (pH 7·3) for 1 h. The material was then dehydrated in increasing concentrations of ethanol cleared in propylene oxide, embedded in epon and cut with diamond knives. Sections about 1 $\mu$m thick were stained with toluidine blue and examined under the light microscope. Ultrathin sections about 50 nm thick were stained with uranyl acetate and lead citrate and examined by transmission electron microscopy (TEM) (Zeiss EM9S).

Peripheral blood cells were fixed in 2% glutaraldehyde in cacodylate buffer (pH 7·3). After 1 h approximately half the material was fixed in osmium and prepared for TEM as described above. The other half was fixed in glutaraldehyde for a further period of 2 h, collected on nuclear pore filters and processed by the critical-point drying method using $CO_2$. The specimens were then examined with a scanning electron microscope (SEM; Cambridge, Stereoscan).

FIG. 4.—Wet-film preparation of peripheral blood from Case 2 showing vermipodia. 850.

FIG. 5.—Wright–Giemsa stained blood smear from Case 2, showing a leukaemic cell (L) with irregular surface ruffling at one pole and surface vacuoles. × 1150.

RESULTS

*Light microscopy*

In the peripheral blood of all cases there were numerous atypical monocytes (histiocytes) with a varying proportion of blasts and immature forms. Blasts predominated in Case 2. Case 3 had the most differentiated forms of monocytes. Erythrophagocytosis was evident in the peripheral blood in Case 1. Cases 1 and 2 showed the most striking and numerous examples of unusual worm-like cellular projections by light microscopy, both in stained peripheral films and in wet preparations (Fig. 4). The cells in all cases showed varying degrees of cell membrane ruffling and irregularities. These were often situated at one pole of the cell, and the cytoplasm immediately underlying these areas contained small blebs or vacuoles (Fig. 5). In the marrows there was an extensive infiltration of neoplastic monocytes, but the unusual cytoplasmic projections were not evident at this site.

*Scanning electron microscopy*

The peripheral blood of all cases showed essentially the same features, but the worm-like projections were most prominent in Case 1 (histiocytic medullary reticulosis). Small cells with short villous processes characteristic of B-type lymphocytes were seen. Numerous larger cells were also evident and these presumably were the leukaemic histiomonocytic cells. They showed quite remarkable variation in their surface characteristics. Some

FIG. 6.—SEM of peripheral blood from Case 1, showing spherical and hemispherical surface blebs (arrow). Note small worm-like process (arrowhead). × 7350.

FIG. 7.—SEM of peripheral blood from Case 1, showing vermipodia (arrows). × 11,600.

FIG. 8.—Blisters seen in leukaemic cells in peripheral blood of Case 3. ×11,000.

FIG. 9.—Appearance here suggests the pinching off of a double-membrane-bound blister and its impending detachment from a leukaemic cell (Case 3). ×26,000.

FIG. 10.—An apparently detached double-membrane-bound blister (Case 3). ×34,500.

were relatively smooth, but many were covered with numerous large hemispherical or spherical projections from their surface (Fig. 6). Even more striking was the presence of long worm-like projections from the cells (Fig. 7). These arose from a small area of the cell surface, were of a relatively constant cross-section and did not have any terminal microspikes. Sometimes they were multiple, the maximum number recorded being 5. They differed from the occasional uropod seen, which was single, with a broad base and tapering body.

FIG. 11.—Multiple vacuoles forming a complex blister at the cell surface (Case 3). × 34,000.

*Transmission electron microscopy*

In the marrows of all cases there was an infiltration of neoplastic cells. Blebs and projections were not prominent, but some were present. The malignant cells in the peripheral blood presented more striking and varied changes, which were again essentially common to all cases. The most prominent abnormality was the presence of numerous small blisters on the surface of the cells (Fig. 8). Such blisters seemed to develop by a single-membrane-bound vacuole approaching the cell surface and becoming covered by another membrane derived from the cell membrane in the process of discharge from the cell (Figs. 9, 10). At times multiple vacuoles formed compound or complex blisters at the cell surface (Fig. 11).

In addition to the clear vacuoles or blisters, multiple projections containing cytoplasmic material (blebs) were also seen (Fig. 12). Here it appeared that by a pinching-off process material was being expelled from the cell in single-membrane-bound structures (Fig. 13), the membrane being derived from the cell membrane. Similarly, vermipodia

FIG. 12.—Cytoplasmic blebs containing ribosomes but no other organelles (Case 3). × 30,800.

FIG. 13.—Appearance seen here suggests the pinching off and discharge of blebs (Case 3). × 30,800.

FIG. 14.—Section through a vermipodium of a leukaemic cell from Case 3. Note absence of larger cell organelles from the vermipodium. × 31,600.

FIG. 15.—Undulating tubules (arrow) in a leukaemic cell from peripheral blood of Case 3. × 43,400.

also contained cytoplasmic material (Fig. 14), but large organelles such as mitochondria or lysosomes were not found here or in the blebs.

Another finding was the occurrence of undulating tubules in the malignant histiocytes of Case 3 (Fig. 15) similar to those described in cases of viral infections, leukaemias, lymphomas and autoimmune diseases such as systemic lupus erythematosus (reviewed by Ghadially, 1975).

### DISCUSSION

Cell processes such as pseudopodia, uropodia and the processes involved in pinocytosis have been well documented (Bessis, 1973; Ghadially, 1975) and their function generally understood. It is not unusual for these to be seen in haemopoietic cells in the peripheral blood. However, the striking and unusual feature in our cases is the occurrence of large numbers of blebs, blisters and worm-like projections which in no way resemble the common phenomena mentioned above.

Review of the literature indicates that the spherical and hemispherical projections seen on the cell surface by SEM would be compatible either with zeiotic blebs (Kessel and Shih, 1974) or the phenomenon described, probably incorrectly, as potocytosis (Zollinger, 1948) or blebbing (Belkin and Hardy, 1961). Our ultrastructural study shows that both phenomena are present, the cytoplasm-filled blebs of zeiosis (Fig. 12) and the clear fluid-filled blisters (Figs. 9, 10). On ultrastructural examination it would appear that the blisters originate in the cytoplasm as vacuoles near the cell surface, which are then discharged from the cell, during which they acquire an additional membranous coat from the cell membrane. The question of the nature of these phenomena, and the situations in which they occur, naturally arises. Their nature will be considered first.

### *Zeiosis*

This term was originally coined to describe the rapid projection and retraction of the cytoplasm of neurons in culture (Costero and Pomerat, 1951) and is now given to the bubbling of the cellular surface as seen in cells in tissue culture, particularly in certain stages o fmitosis (Price, 1967; Porter, Prescott and Frye, 1973). It can also be induced by certain agents having an action on the microfilaments, such as cytochalasin D (Miranda *et al.*, 1974; Godman *et al.*, 1975). Ultrastructural studies (Price, 1967) by TEM show that zeiotic blebs are cytoplasm-filled protuberances, generally without large cell organelles. The cause of this phenomenon is not known for certain, but is thought to be related to the cytomembrane system (Rose, 1964) and action or abnormal function of the microfilaments (Godman *et al.*, 1975).

*" Blisters "*

By light and scanning electron microscopy this phenomenon is difficult to distinguish from zeiosis, but TEM demonstrates that in blistering the protuberances are filled with clear fluid, and not cytoplasmic material as is the case in zeiosis. Clear fluid-filled blisters have been reported to occur in malignant cells spontaneously, and in normal and malignant cells on exposure to certain heavy metals (Belkin and Hardy, 1961) and it is thought that it is due to the action of these on the sulphydryl groups of the cell membrane. It was also noted (Belkin and Hardy, 1961) that not all types of cell would blister on exposure to these agents, and that malignant cells were more prone to show this phenomenon than normal ones. Formation and release of a large number of membrane-bound vacuoles have also been seen on exposure of cultured human leukaemic lymphoblasts to vinblastine and vincristine (Krishnan and Frei, 1975). The mode of action of the vinca alkaloids in this phenomenon is not known, but is thought to be related to their effect on the cell membrane, either by affecting membrane transport or from their effect on the cytoplasmic microtubules. It is again interesting to note that in this experiment (Krishnan and Frei, 1975) it was only the leukaemic blast cells which showed this phenomenon, and not fibroblasts growing in culture; thus confirming that both the correct *milieu* and cell type are essential for the production of this phenomenon.

*Worm-like processes or vermipodia*

We have briefly described the scanning and light microscopic features of these processes (Ghadially and Skinnider, 1977) but to our knowledge they have not been otherwise reported previously. These slender processes arise from a small area of the cell surface, have a constant cross-sectional area and are often multiple (Fig. 7). They thus differ from uropods which are broad-based, tapering, single processes often ending in microspikes. The function and significance of vermipodia are not clear. One may hypothesize that they have a locomotor function, or that they may serve to explore contact with other cells, as has been suggested for uropodia. However, there is no concrete evidence to support either of these ideas, and it may be that as they arise in malignant cells they are only pathological alterations of the surface with little functional significance.

*Significance of cell surface changes*

Why these various phenomena (*i.e.* the formation of blebs, blisters and vermipodia) should be seen in malignant histiocytosis and a case of leukaemic reticulum-cell sarcoma is not clear. They cannot be dismissed as artefacts of tissue preparation for they were seen after various preparative procedures. Thus they were seen in routine Wright–Giemsa-stained peripheral films, in wet-field preparations and by TEM and SEM. Further, the method of preparation of the material for ultrastructural examination was the same as that for many other cases of haemopoietic leukaemias and lymphomas which we have studied (Ghadially and Skinnider, 1972; Skinnider and Ghadially, 1973; Ghadially and Skinnider, 1974; Skinnider and Ghadially, 1975) and in which no similar abnormality was evident. Neither can it be attributed to the agonal activity of a dying cell, as the nucleus and cytoplasmic organelles show no evidence of serious damage. As two of the patients had been exposed to vincristine, it is tempting to implicate this in the production of the blebs and blisters, as this effect of vincristine has been demonstrated *in vitro* (Krishnan and Frei, 1975). However, such an hypothesis is not supported by the fact that cell surface changes were quite prominent in Case 3, in which the only drug given was prednisone. However, drug action cannot be excluded. The one thing the cases have in common is the presence of malignant histiocytes circulating freely in the peripheral blood. As Belkin and Hardy (1961) point out, " every blebbing cell has been a malignant cell, or a normal cell grown in tissue culture, *i.e.* as a free or unattached cell and not part of a solid aggregate ". It may thus be that the cells of malignant histiocytosis are so prone to develop this phenomenon that they spontaneously develop blebs and blisters when circulating in the peripheral blood. The fact that all the cases in which these abnormalities were seen have neoplastic histiocytes circulating in the blood would indicate that the susceptibility to these phenomena lies mainly in the neoplastic cell. The importance of cell surface changes as a marker of malignant transformation is being increasingly recognized (Nicholson, 1976; Nicholson and Poste, 1976*a*; Nicholson and Poste, 1976*b*) and it may be that these morphological changes are one expression of the complex alterations in the cell surface of these malignant histiocytes.

This work was supported by a grant from the National Cancer Institute of Canada and from the Medical Research Council of Canada. We wish to thank Dr W. Khan, who kindly supplied the material for Case 3.

### REFERENCES

BELKIN, M. & HARDY, W. G. (1961) Relation Between Water Permeability and Integrity of Sulfhydryl Groups in Malignant and Normal Cells. *J. Biophys. Biochem.*, **9**, 733.

BESSIS, M. (1973) *Living Blood Cells and Their Ultrastructure.* New York, Heidelberg, Berlin: Springer Verlag.

CLINE, M. J. & GOLDE, D. W. (1973) A Review and Re-evaluation of the Histiocytic Disorders. *Am. J. Med.*, **55**, 49.

COSTERO, I. & POMERAT, C. M. (1951) Cultivation of Neurons from the Adult Human Cerebral and Cerebellar Cortex. *Am. J. Anat.*, **89**, 405.

GHADIALLY, F. N. (1975) *The Ultrastructural Pathology of the Cell.* London: Butterworth.

GHADIALLY, F. N. & SKINNIDER, L. F. (1972) " Hairy " Cell Leukemia—An Ultrastructural Study. *Cancer, N.Y.*, **29**, 444.

GHADIALLY, F. N. & SKINNIDER, L. F. (1974) Giant Mitochondria in Erythroleukemia. *J. Path.*, **114**, 113.

GHADIALLY, F. N. & SKINNIDER, L. F. (1976) Vermipodia—a new type of cell process. *Experientia*, **32**, 1061.

GODMAN, G. C., MIRANDA, A. F., DEITCH, A. D. & TANENBAUM, S. W. (1975) Action of Cytochalasin D on Cells of Established Lines. *J. Cell Biol.*, **64**, 644.

KESSEL, R. G. & SHIH, C. Y. (1974) *Scanning Electron Microscopy in Biology.* New York, Heidelberg, Berlin: Springer Verlag.

KRISHNAN, A. & FREI, E. (1975) Morphological Basis for the Cytolytic Effect of Vinblastine and Vincristine on Cultured Human Leukaemic Lymphoblasts. *Cancer Res.*, **35**, 497.

MIRANDA, A. F., GODMAN, G. C., DEITCH, A. D. & TANENBAUM, S. W. (1974) Action of Cytochalasin D on Cells of Established Lines. *J. Cell Biol.*, **61**, 481.

NICHOLSON, G. L. (1976) Trans-membrane Control of the Receptors on Normal and Tumor Cells. II. Surface Chances Associated with Transformation and Malignancy. *Biochim. biophys. Acta*, **458**, 1.

NICHOLSON, G. L. & POSTE, G. (1976*a*) The Cancer Cell: Dynamic Aspects and Modifications in Cell-surface Organization. I. *New Engl. J. Med.*, **295**, 197.

NICHOLSON, G. L. & POSTE, G. (1976*b*). The Cancer Cell: Dynamic Aspects and Modifications in Cell-surface Organization. Pt. II. *New Engl. J. Med.*, **295**, 253.

POLLIACK, A., MCKENZIE, S., GEE, T., LAMPEN, N., HARVEN, E. & CLARKSON, B. D. (1975) A Scanning Electron Microscopic Study of 34 Cases of Acute Granulocytic, Myelomonocytic, Monoblastic and Histiocytic Leukaemia. *Am. J. Med.*, **59**, 308.

PORTER, K. R., PRESCOTT, D. & FRYE, J. (1973) Changes in Surface Morphology of Chinese Hamster Ovary Cells During the Cell Cycle. *J. Cell Biol.*, **57**, 815.

PRICE, Z. H. (1967). The Micromorphology of Zeiotic Blebs in Cultured Human Epithelial (HEp) Cells. *Expl Cell Res.*, **48**, 82.

ROSE, G. G. (1964) Phase Contrast Microscopy in Living Cells. *J. R. microscop. Soc.*, **83**, 97.

SCHNITZER, B. & KASS, L. (1973) Leukemic Phase of Reticulum Cell Sarcoma (Histiocytic Lymphoma). A Clinicopathologic and Ultrastructural Study. *Cancer, N.Y.*, **31**, 547.

SCHUMACHER, H. R., SZEKELY, I. E. & PARK, S. A. (1973) Monoblast of Acute Monoblastic Leukemia. *Cancer, N.Y.*, **31**, 209.

SKINNIDER, L. F. & GHADIALLY, F. N. (1973) Glycogen in Erythroid Cells. *Arch. Path.*, **92**, 139.

SKINNIDER, L. F. & GHADIALLY, F. N. (1975) Ultrastructure of Acute Myeloid Leukemia Arising in Multiple Myeloma. *Human Path.*, **6**, 379.

WILLIAMS, W. J. (1973) *Haematology.* Toronto McGraw Hill.

ZAWADSKI, Z. A., PENA, C. E. & FISHER, E. R. (1969) Histiocytic Medullary Reticulosis. *Acta Haemat.*, **42**, 50.

ZOLLINGER, H. U. (1948) Cytologic Studies with the Phase Microscope I. The Formation of " Blisters " on Cells in Suspension (Potocytosis), with Observations on the Nature of the Cellular Membrane. *Am. J. Path.*, **24**, 545.